# Impact of *Escherichia coli* Outer Membrane Vesicles on Sperm Function

**DOI:** 10.3390/pathogens11070782

**Published:** 2022-07-10

**Authors:** Veronica Folliero, Marianna Santonastaso, Federica Dell’Annunziata, Pasquale De Franciscis, Giovanni Boccia, Nicola Colacurci, Anna De Filippis, Massimiliano Galdiero, Gianluigi Franci

**Affiliations:** 1 Department of Experimental Medicine, University of Campania Luigi Vanvitelli, 80138 Naples, Italy; veronica.folliero@unicampania.it (V.F.); federica.dellannunziata@unicampania.it (F.D.); anna.defilippis@unicampania.it (A.D.F.); massimiliano.galdiero@unicampania.it (M.G.); 2 Department of Woman, Child and General and Special Surgery, University of Campania Luigi Vanvitelli, 80138 Naples, Italy; marianna.santonastaso@unicampania.it (M.S.); pasquale.defranciscis@unicampania.it (P.D.F.); nicola.colacurci@unicampania.it (N.C.); 3 Department of Medicine, Surgery and Dentistry Scuola Medica Salernitana, University of Salerno, 84131 Baronissi, Italy; gboccia@unisa.it

**Keywords:** outer membrane vesicle, fertility, *Escherichia coli*

## Abstract

Reproductive tract infections account for approximately 15% of male infertility cases. *Escherichia coli* (*E. coli*) represents the most frequently isolated bacterial strain in the semen of infertile men. All Gram-negative bacteria constitutively produce outer membrane vesicles (OMVs). The present study proved, for the first time, the involvement of OMVs in human sperm function. *E. coli* OMVs were isolated by ultracentrifugation and characterized via sodium dodecyl sulfate–polyacrylamide gel electrophoresis (SDS-PAGE), transmission electron microscopy (TEM) and dynamic light scattering (DLS) analysis. Human sperm was exposed to OMVs (8 µg/mL) for different times (30, 45, 60 and 90 min). The vitality, motility, morphology, ROS level and DNA fragmentation of spermatozoa were evaluated. OMVs reduced the progressive motility and increased the immobile spermatozoa amount after 30 min of treatment. In addition, a significant increase in the percentage of intracellular ROS and sperm DNA fragmentation was recorded for each vesicular exposure time. These preliminary findings prove that OMVs contribute to altering human sperm function via two mechanisms: (i) impaired motility and (ii) DNA fragmentation.

## 1. Introduction

Infertility is a highly widespread disease affecting between 60 and 168 million people worldwide [1]. The World Health Organization (WHO) defines infertility as the inability to achieve a successful pregnancy after 12 months following appropriate and unprotected sexual intercourse or therapeutic donor insemination [2]. Approximately 50% of infertility cases are ascribed to males for several conditions, such as: (i) hormonal alteration, (ii) ejaculatory failure, (iii) varicocele, (iv) obstructive problems, (v) cryptorchidism and (vi) male reproductive tract infections (MRTIs) [3]. MRTIs account for 6–10% of male infertility cases [4]. These infections cause qualitative and quantitative alterations in sperm parameters, leading to serious problems in male reproductive health [5]. Bacteria represent the main cause of MRTIs, although viruses, fungi and parasites are involved in the infection process. *Staphylococcus epidermidis*, *Streptococcus viridans*, *Staphylococcus aureus*, *Enterococcus faecalis* and *E. coli* are the most frequently isolated bacterial agents from semen samples of patients with compromised sperm parameters [6].

*E. coli* causes 65–80% of bacterial prostatitis cases. Among the several pathogenic strains of *E. coli*, uropathogens belonging to serotypes O1, O2, O4 and O6 are often involved in infection of the male reproductive tract [7,8]. Several studies have reported the effect of *E. coli* on sperm function. Contact between bacteria and spermatozoa leads to a decrease in sperm motility, mainly due to the agglutinating effect of the sperm. Monga and Roberts showed that this process is induced by fimbria types 1 and P of *E. coli* [9]. Type 1 results in head-to-head spermatozoa interaction through bonds between mannose residues on the surface of the sperm head. Indeed, fimbria type P causes head–tail agglutination using galactosyl residues exposed throughout the spermatozoa [10,11,12]. The decrease in sperm motility is also due to the reduction in the mitochondrial membrane potential of spermatozoa following contact with pathogens [13]. A study by Kushawaha et al. revealed that *E. coli* induces damage to the sperm head and compromises the acrosome, leading to cell death and impaired acrosome reaction [14]. Other important effects of *E. coli* on sperm are the increase in intracellular reactive oxygen species (ROS) levels and DNA fragmentation [14]. The role of *E. coli* in male infertility is widely known, whereas no studies have been conducted on *E. coli*-derived OMV effects on human sperm function.

OMVs are spherical lipid bilayer nanostructures with a diameter of 20–250 nm and are constitutively released by the bacterium into the host environment [15]. These vesicles consist of outer membrane components, such as lipopolysaccharide (LPS), phospholipids and outer membrane proteins, and periplasmic and cytoplasmic fractions, including genetic material and virulence factors such as invasion and evasion proteins and toxins [16]. OMVs are involved in numerous functions that are protective for the bacterium itself and offensive to the host, promoting pathogenesis. These vesicles are involved in nutrient acquisition, biofilm formation, gene transfer, and the delivery of toxins, adhesins and immunity modulators [17].

To date, the impact of *E. coli* OMVs on human sperm function has not yet been evaluated. Knowledge of all of the mechanisms by which *E. coli* alters sperm function will allow the improvement of intervention strategies to avoid the consequences of MRTIs.

## 2. Results

### 2.1. Identification of OMVs Derived from E. coli

**Figure 1:**
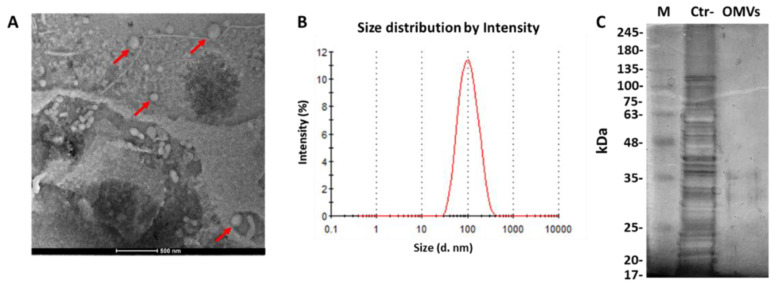
Characterization of OMVs derived from *E. coli* ATCC 700928. (**A**) OMVs visualized by TEM; (**B**) vesicular diameter via DLS analyses; (**C**) silver-stained SDS-PAGE (10%) protein profiles of *E. coli* ATCC 700928 and related OMVs; molecular mass marker (M) is expressed in kilodalton (kDa).

To purify *E. coli-*derived OMVs, sequential filtrations and ultracentrifugation were conducted on bacteria-free cultures. To ensure accurate analysis, three independent vesicular purifications were performed. The amount of OMVs was determined based on the protein yield. Protein concentrations of 84 ± 0.05 µg/mL were obtained from 500 mL of bacterial culture. TEM analysis detected spherical vesicles, and no bacterial contamination was observed (Figure 1A). The vesicular diameter and size distribution were examined through DLS. DLS data showed that most of the vesicles were 98.4 ± 1.1 nm in diameter and were characterized by heterogeneous size distribution, proven by a PDI of 0.229 ± 0.024 (Figure 1B). The protein profile of *E. coli* OMVs was obtained from 5 µg of proteins via 10% SDS-PAGE. In contrast to bacterial extract, two major bands in the range of 30–40 KDa were detected in *E. coli* OMVs, proving the absence of bacterial contaminants in the vesicular purification (Figure 1C).

### 2.2. Effect of E. coli OMVs on Sperm Quality

To analyze the effects of the vesicles on sperm quality, purified spermatozoa were treated with 8 µg/mL OMVs for 30, 45, 60 and 90 min, and sperm motility, viability and morphology were evaluated. Compared to the untreated spermatozoa (negative control, CTR−), no significant difference was observed in the rate of viability and morphology (*p*-value > 0.5). As well as benzene (positive control, CTR+), OMVs significantly reduced motility and increased the rate of immobile spermatozoa. Serious impairment of normal sperm function was observed only 45 min after vesicular treatment. In particular, rates of sperm mobility and immobile spermatozoa were 51 and 49% after OMV treatments, compared to 64 and 36% in the control group, respectively (*p*-value < 0.05). After 60 min of treatment, sperm motility decreased by 12% (*p*-value < 0.05). The largest effect was achieved after 90 min of vesicular exposure, recording levels of sperm mobility and immobile spermatozoa of 39 and 61%, compared to 60 and 42% in untreated spermatozoa (*p*-value < 0.05) (Table 1).

### 2.3. Assessment of Intracellular ROS Levels

**Figure 2:**
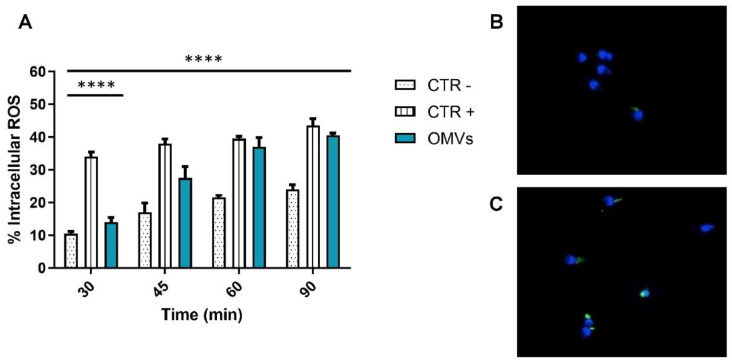
(**A**) Percentage of intracellular ROS in sperm after OMV exposure for 30, 45, 60 and 90 min. The black bars represent the CTR− (sperm treated with PBS1X); the dark gray bars are CTR+ (sperm treated with 0.4 µL/mL of benzene); and the light gray bars are 8 μg/mL OMV-treated sperm. (**B**) Intracellular ROS (green color) in sperm cells were analyzed by fluorescence microscopy with 100× magnification equipped with BP 330–380 nm and LP 420 nm filters after solvent exposure for 45 min. (**C**) Intracellular ROS (green color) in sperm cells were analyzed by fluorescence microscopy with 100× magnification equipped with BP 330–380 nm and LP 420 nm filters after 8 μg/mL OMV exposure at 45 min. ****: *p*-value < 0.0001.

The ability of OMVs to induce the formation of ROS was evaluated through the DCF assay. OMV treatments caused significant ROS accumulation (intense green fluorescence) in the spermatic neck region. Data indicated that the rates of sperm with DCF fluorescence were significantly higher than those associated with the negative control groups (*p*-value < 0.0001). Compared with the control groups, relative ROS levels increased following OMV treatments in a time-dependent manner. The sperm intracellular ROS rates were significantly high after only 45 min of OMV treatment, recording a 17.5% increase compared to untreated spermatozoa. After 60 and 90 min of vesicular treatment, ROS levels were comparable to those of the positive control. In particular, ROS percentages were 35.5 and 40.5%, compared to 21.5 and 24% in the negative control group, after 60 and 90 min of treatment, respectively (*p*-value < 0.0001) (Figure 2).

### 2.4. Evaluation of Sperm DNA Fragmentation

**Figure 3:**
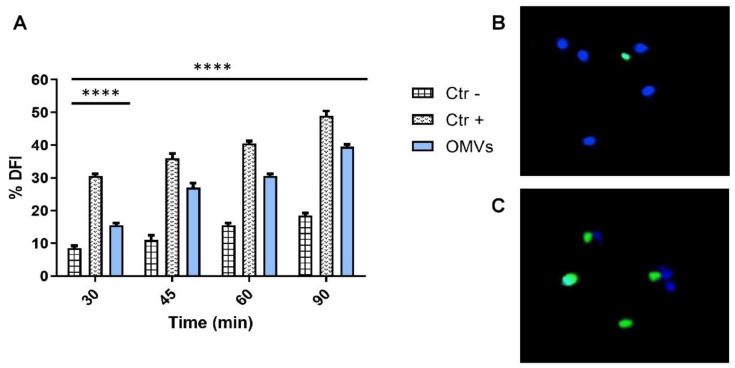
(**A**) DFI (%) in sperm after OMV exposure for 30, 45, 60 and 90 min. The black bars represent CTR− (sperm treated with PBS1X); the dark gray bars are CTR+ (sperm treated with 0.4 µL/mL of benzene); and the light gray bars are 8 μg/mL OMV-treated sperm. (**B**) Sperm with fragmented DNA (green) and undamaged DNA (blue) after solvent exposure for 45 min detected by fluorescence microscopy with 100× magnification equipped with BP 330–380 nm and LP 420 nm filters after solvent exposure and (**C**) treatment with 8 μg/mL OMVs. ****: *p*-value < 0.0001.

Sperm DNA damage was evaluated through the TUNEL assay. OMV treatments induced significant DNA fragmentation (intense green nuclear fluorescence) in a time-dependent manner. The DNA fragmentation cut-off (26%) was exceeded after 45 min of vesicular exposure, recording a DFI (DNA fragmentation index) of 27%. After 60 min of OMV treatment, DFI rates increased by 15% in treated spermatozoa compared to untreated ones. A greater impact was observed after 90 min of vesicular exposure. In particular, 39.5 and 18.5% DFIs were obtained for OMV-treated and untreated spermatozoa, respectively (Figure 3).

OMV exposure for 45 min was detected by fluorescence microscopy with 100× magnification equipped with BP 330–380 nm and LP 420 nm filters after solvent exposure.

## 3. Discussion

MRTIs represent up to 15% of male infertility cases worldwide. Chronic infections and the consequent inflammatory events induce qualitative and quantitative alterations in the sperm, compromising its fertilizing potential. Several studies reported that bacteria cause sperm dysfunction. These microorganisms directly affect male reproductive function through the agglutination of motile spermatozoa and impaired acrosome reaction and, indirectly via ROS production, cause DNA and membrane damage, cellular death and a reduction in sperm motility. The bacteria responsible for MRTIs generally derive from the urinary tract or are transmitted to partners through sexual intercourse. Uropathogenic *E. coli* represents one of the main microorganisms isolated in the semen of infertile patients. The pathogenic action of this strain is associated with the expression of a wide variety of virulence factors, including fimbria types 1 and P, LPS, non-hemagglutinin adhesin-siderophore receptor, salmochelin siderophore receptor, alpha-hemolysin toxin and outer membrane protease T. *E. coli*, like all Gram-negative bacteria, constitutively release OMVs in the host environment. These vesicles contribute to bacterial pathogenesis, acting as vectors of virulence factors. Much evidence suggests that OMVs induce a strong proinflammatory immune response in the host through the interaction between pathogen-associated molecular patterns and toll-like receptors, inducing the secretion of cytokines or proinflammatory receptors [18]. 

To date, the effects of *E. coli* OMVs on human sperm have not yet been reported. Here, some of the mechanisms by which *E. coli* affects sperm function through the secretion of OMVs are shown. In this study, the viability, motility and morphology of spermatozoa were initially assessed after OMV exposure. Sperm morphology and viability were not significantly affected by vesicular treatments. Gao et al. reported that the lack of the detection of morphological alterations in spermatozoa could be due to the masking of the sperm surface by OMVs, which induce signal transduction mechanisms through interactions with surface receptors [19]. Regarding sperm viability, no effect was detected following vesicular exposure. Villegas et al. demonstrated that apoptotic sperm death was detected 60 min after exposure with 60 × 10^6^ CFU/mL of *E. coli* strains [20]. In our study, OMVs did not contribute to the induction of cell death, probably because the selected times and vesicular concentration of treatments are not sufficient to observe sperm death. Regarding motility, it was strongly influenced by OMVs, observing significant impairments after 45 min of treatment. The sperm motility impairment may be a consequence of high ROS production.

ROS have an important role in spermatic function and fertilization. A correct balance of ROS and antioxidants is essential for the acrosome reaction, motility and binding of the sperm membrane to zona pellucida glycoprotein 3, promoting sperm–oocyte fusion [21]. Though ROS are required for regular physiological sperm function, excessive oxidative stress impairs sperm function, leading not only to infertility but also to abortions and hereditary genetic mutations in the child [22]. In this study, intracellular sperm ROS levels were significantly higher after only 45 min of OMV treatments compared to untreated spermatozoa. High ROS levels were also recorded after treating boar sperm with 5 μg/mL Proteus mirabilis OMVs, according to the study by Gao et al. [19]. Oxidative stress induces lipid oxidation, resulting in extensive sperm membrane damage. The by-products of lipid oxidation led to reduced sperm motility and DNA damage. Morielli et al. proved that the decrease in motility was due to ROS-induced peroxidation of lipids in the sperm membrane, which decreases cellular flexibility, and to the inhibition of motility mechanisms [23]. Furthermore, Gualtieri et al. showed that ROS-induced mitochondrial damage resulted in a decrease in ATP, impeding sperm motility [24].

In our study, OMV treatment caused a significant increase in DNA fragmentation that exceeded the DNA fragmentation cut-off of 26%. This could be a consequence of the free radical production that induces damage to sperm DNA through single- and double-strand DNA breaks, cross-links and chromosomal rearrangements [25]. DNA damage is also mediated by caspases in the ROS-induced apoptosis process [26].

Overall, our preliminary results prove that OMVs derived from uropathogenic *E. coli* contribute to sperm dysfunction via two mechanisms: (i) impaired motility and (ii) sperm DNA fragmentation. OMV-induced oxidative stress represented the triggering factor of sperm damage. Further investigations are needed to better understand the molecular mechanism underlying sperm damage.

## 4. Materials and Methods

### 4.1. Bacterial Strain and Growth Conditions

*E. coli* ATCC 700928 was obtained from the American Type Culture Collection (ATCC, USA) and used for OMV purification. *E. coli* was grown in Luria–Bertani (LB) medium (Difco, Sparks, MD, USA) at 37 °C in aerobic conditions with orbital shaking (180 rpm) [27].

### 4.2. OMV Purification

*E. coli* was grown in LB broth (500 mL, 37 °C, 180 rpm) until reaching an OD600 nm value of 1. Bacterial cells were removed through centrifugation at 3000× *g* for 20 min at 4 °C. Residual bacteria and cellular debris were eliminated by sequential 0.45 and 0.22 µm filtration (VWR Scientific, Bridgeport, NJ, USA). OMVs were collected by centrifugation at 100,000× *g* for 2 h at 4 °C (Optima XPN-100 Beckman Coulter and rotor 70Ti, Palo Alto, CA, USA). The vesicles were washed in sterile PBS1X through ultracentrifugation and re-suspended in 150 μL of PBS1X. LB agar plates were used to check the sterility of the OMVs. The purified vesicles were stored at −20 °C until use [28].

### 4.3. Dynamic Light Scattering (DLS) Analysis

The Z-average size (Z-ave) and the polydispersity index (PDI) of the OMVs were obtained by DLS analysis using Malvern Zetasizer ZS90 (Malvern Panalytical Ltd., Malvern, UK). Z-ave indicates the mean diameter of the vesicles, while PDI defines the vesicular distribution according to the size. Three independent measurements were performed for each of the three independent OMV purifications. The obtained data were analyzed using Dispersion Technology Software (DTS) (V7.01). The analysis provided the vesicular diameter in nm (d. nm) and the PDI. PDI values below 0.05 indicated a monodisperse vesicular population, while values above 0.2 were associated with samples with a wide vesicular distribution.

### 4.4. Transmission Electron Microscopy (TEM)

TEM was used to detect the morphology of OMVs. The OMV sample was deposited on a formvar carbon-coated copper grid. After adsorption of the sample, the grid was washed with PBS1X and negatively stained with Uranyl Acetate (*w*/*v* 1%) (Electron Microscopy Science, Hatfield, PA, USA). The images were obtained using FEI TECNAI G2 S-twin 200 kV apparatus operating at 120 kV.

### 4.5. Sodium Dodecyl Sulfate–Polyacrylamide Gel Electrophoresis (SDS-PAGE)

The amount of *E. coli* OMVs was measured by the Bradford assay (Bio-Rad Laboratories, Hercules, CA, USA). The protein profile of *E. coli* and related OMVs was obtained from 5 µg of proteins via 10% SDS-PAGE and displayed with silver staining. A protein molecular weight standard from 35 to 180 KDa was used (Bio-Rad Laboratories, Hercules, CA, USA).

### 4.6. Human Semen Sample Collection and Purification

Semen samples were collected from men aged 26 to 37 through masturbation after 3–5 days of abstinence at the reproduction biology laboratory in University Hospital Luigi Vanvitelli. Three semen samples with suitable parameters, in accordance with the WHO 2010 guidelines, were selected for the treatments (Table 2) (https://www.who.int/docs/default-source/reproductive-health/srhr-documents/infertility/examination-and-processing-of-human-semen-5ed-eng.pdf, 21 April 2021). After liquefaction at room temperature for 30 min, spermatozoa were purified by 45–90% double density gradient centrifugation, according to the manufacturer’s instruction (SPERM GRAD™; Vitrolife, Göteborg, Sweden). The pellet was dissolved in a culture medium (MEM buffered with HEPES; Sigma Aldrich^®^, Milan, Italy) to a final concentration of 4 × 10^6^ spermatozoa/mL.

### 4.7. Exposure to OMVs and Semen Parameter Evaluation

Each purified sample was divided into 4 aliquots (1 × 10^6^ spermatozoa/mL) of 600 μL volume: (i) untreated sample; (ii) sample treated with PBS1X (150 µL) as CTR−; (iii) sample treated with 0.4 µL/mL of benzene as CTR+; and (iv) sample treated with 8 µg/mL OMVs (150 µL). The treatments were conducted in a culture medium at 37 °C for 30, 45, 60 and 90 min under agitation. After treatments, samples were centrifuged for 5 min at 1500 rpm, pellets were dissolved in 1 mL of culture medium (MEM buffered with HEPES; Sigma Aldrich^®^, Milan, Italy) and the semen parameters were assessed. Sperm concentration and motility were evaluated by loading 20 µL of sample into the Makler chamber by optical phase-contrast microscope at 200× magnification (Zeiss, New York, NY, USA). Total motility, including the totality of progressive and non-progressive sperm, was assessed by counting approximately 200 spermatozoa in a total of at least 5 fields in each replicate (https://apps.who.int/iris/handle/10665/44261 accessed on 1 May 2022). Normal sperm morphology was assessed by Test-simplets^®^prestained slides (Origio, Cooper Surgical, Inc., Trumbull, CT, USA), while sperm vitality was evaluated via eosin–nigrosin staining.

### 4.8. Dichloro-Dihydro-Fluorescein Diacetate (DCFH-DA) Test

Intracellular levels of ROS were detected using DCFH-DA (Thermo Fisher Scientific, Waltham, MA, USA). The latter is de-esterified to DCFH, which is oxidized to fluorescent DCF, in the presence of ROS. The sperm pellets obtained from 150 μL of the sample were incubated with 13 μM DCFH2-DA at 37 °C for 30 min. Subsequently, the samples were counterstained with DAPI for 5 min and placed on a glass slide for analysis with a fluorescence microscope equipped with BP 330–380 nm and LP 420 nm filters (Nikon Eclipse E-600, Tokyo, Japan). The percentage of intracellular ROS was visually counted and evaluated as the percentage of spermatozoa showing a positive response (green cells) to the total sperm. The DCFH-DA assay was performed in triplicate [29].

### 4.9. Terminal Deoxynucleotidyl Transferase (TdT)dUTP Nick-End Labeling (TUNEL) Assay

Sperm DNA fragmentation was evaluated using In Situ Cell Death Detection Kit (Roche Diagnostics, Basel, Switzerland) according to the manufacturer’s instructions. Briefly, 15 µL of ejaculate was fixed with 4% paraformaldehyde and air-dried. The samples were put on glass slides and incubated in a permeabilizing solution (0.1% Triton X-100 in 0.1% sodium citrate) for 2 min at 4 °C. After washing in a bicarbonate buffer, the TUNEL reaction mix (5 µL of terminal deoxynucleotidyl transferase enzyme solution and 45 µL of label solution) was added to both glass slides and incubated in a humid chamber at 37 °C for 1 h. Then, the samples were stained for 5 min with 4′,6-Diamidine-2′-phenylindole dihydrochloride (DAPI) solution, and, after, 100 µL of 1,4 diazobicyclo (2,2,2) octane (DABCO) solution (20×) was added. The glass slides were examined by a fluorescence microscope equipped with BP 330–380 nm and LP 420 nm filters (Nikon Eclipse E-600, Tokyo, Japan), and the percentage of fragmented sperm DNA was reported as the percentage of TUNEL-positive sperm. The TUNEL test was performed in triplicate [30].

### 4.10. Statistical Analysis

Sperm parameters, DFI and intracellular ROS levels were expressed as the mean ± standard deviation (SD). The significance of the difference between treated samples and CTR− was obtained via one-way analysis of variance (ANOVA) by the software Graph Pad Prism 6.0 (San Diego, CA, USA). The effect was considered significant at *p*-value < 0.05 [31].

## Figures and Tables

**Table 1:** Sperm vitality and motility after OMV treatment.

OMV–Solvent Concentration	Time of Treatment (min)	Vitality (%)	Motility (P + NP) (%)	Immobile (%)	Normal Morphology (%)
8 µg/mL	30	70.5 ± 0.71 ^●^	54.5 ± 1.4 **	45.5 ± 3.5 ^□□^	19.5 ± 2.1 ^●^
	45	72 ± 1.41 ^●^	51 ± 2.8 ***	49 ± 0.71 **	19 ± 0.70 ^●^
	60	66.5 ± 1.32 ^●^	42.5 ± 0.71 ^▪▪▪^	57.5 ± 6.1 ***	18.5 ± 4.9 ^●^
	90	64 ± 0.89 ^●^	39 ± 0.71 ^◦◦◦^	61 ± 2.8 ^◊◊◊^	17 ± 2.8 ^●^
CTR−	30	71.5 ± 0.78	65.5 ± 6.36	34.5 ± 2.8	21 ± 1.9
	45	71.3 ± 0.35	64 ± 1.4	36 ± 2.1	20.5 ± 1.4
	60	66 ± 1.39	63 ± 2.1	37 ± 1.4	19.5 ± 1.23
	90	64.5 ± 2.12	60 ± 2.8	42 ± 1.3	18 ± 2.6
CTR+	30	65.5 ±2.2 *	48 ± 5.40 ***	52 ± 1.2 ***	18 ± 1.3 ^●^
	45	59 ± 8.6 **	42 ± 6.80 ^✩✩✩^	58 ± 3.4 ^△△△^	16 ± 2.2 **
	60	48 ± 4.5 ***	35 ± 4.40 ****	65 ± 1.5 ^▲^^▲▲^	14 ± 3.5 ^⬢⬢^
	90	40 ± 2.4 ^§§§^	27 ± 1.50 ^▽▽▽^	71 ± 3.2 ****	13 ± 0.60 ^##^

OMVs vs. CTR−. Vitality, ●: not significant; Motility, **: *p*-value = 0.0012, ***: *p*-value = 0.0004, ▪▪▪ *p*-value = 0.0003, ^◦◦◦^: *p*-value = 0.0002; Immobile, ^□□^: *p*-value = 0.0041, **: *p*-value = 0.0012, ***: *p*-value = 0.0001, ^◊◊◊^: *p*-value = 0.0002. CTR+ vs. CTR−. Vitality, *: *p*-value = 0.0136, **: *p*-value = 0.0034, ***: *p*-value = 0.0002, ^§§§^: *p*-value = 0.0002; Motility, ***: *p*-value = 0.0002, ^✩✩✩^ *p*-value = 0.0002, ****: *p*-value < 0.0001, ^▽▽▽^: *p*-value = 0.0001; Immobile, ***: *p*-value = 0.0002, ^△△△^: *p*-value = 0.0003, ^▲▲▲^: *p*-value = 0.0002, ****: *p*-value < 0.0001; Normal morphology, ^●^: not significant, **: *p*-value = 0.0074, ^⬢⬢^: *p*-value = 0.0073, ^##^: *p*-value = 0.0096.

**Table 2:** Parameters of semen samples for the treatments.

Sperm Parameters	Mean ± SD
Semen volume (mL)	3.1 ± 0.42
pH	7.5 ± 0.21
Sperm concentration (10^6^ sperm/mL)	60 ± 83.6
Vitality (%)	76 ± 2.83
Progressive motility (%)	71 ± 2.75
Non-progressive motility (%)	15 ± 1.89
Immobile (%)	19 ± 3.32
Normal morphology (%)	21 ± 2.8

## Data Availability

The data presented in this study are available on request from the corresponding author.
